# The Role of Non-Steroidal Anti-Inflammatory Drugs in the Chemoprevention of Breast Cancer

**DOI:** 10.3390/ph3051550

**Published:** 2010-05-17

**Authors:** Sarah L. Horn, Ian S. Fentiman

**Affiliations:** Hedley Atkins Breast Unit, Guy’s Hospital, London SE1 9RT, UK; E-Mail: Ian.Fentiman@gstt.nhs.uk

**Keywords:** non-steroidal anti-inflammatory drugs, breast cancer, chemoprevention

## Abstract

Epidemiological evidence suggests that non-steroidal anti-inflammatory drugs (NSAIDs) which act as cyclooxygenase (COX-2) inhibitors may reduce breast cancer incidence by up to 20%. These agents are often taken for pain relief by older women with osteoarthritis. Age is the major risk factor for breast cancer in women with 50% cases being diagnosed in those aged >65 years. NSAIDs reduce serum estradiol by 17% in post-menopausal women and since most of these who develop breast cancers have estrogen receptor positive tumours; this suggests a possible preventative role. Careful use of these agents could provide a strategy for both relief of symptoms of osteoarthritis and also breast cancer prevention. Instead of conducting a randomised trial, proof of efficacy could be from an adequately powered cohort study within the breast screening programme.

## 1. Introduction

Chemoprevention is the use of a drug or intervention to interfere with pathogenesis and thereby prevent disease. In the last 20 years NSAIDS with COX-2 inhibition have shown potential in the chemoprevention of many forms of solid malignant neoplasms, including cancers of the colon, breast, lung and prostate.

Breast cancer is the most common malignancy in women in developed countries, with over half the cases being diagnosed in those over 65 years, and it is this older population that represents the fastest growing population group in Europe. The diagnosis can be psychologically devastating and thus any mechanism to reduce risk has a hugely important role to play in terms of both morbidity and mortality. The mainstay of breast cancer treatment is surgical intervention with chemotherapy, radiotherapy and endocrine manipulation, either in the neo-adjuvant and/or adjuvant setting. Drug treatment takes the form of tamoxifen directly acting on estrogen receptors (ER) in premenopausal women or the aromatase Inhibitors, letrozole and anastrazole, acting on the pathways causing inhibition of peripheral estrogen synthesis in the postmenopausal. To date, the main chemo preventative strategy has been the use of endocrine manipulation. 

In 1986 Cuzick *et al.* [1] laid out the theoretical basis for the prevention of breast cancer using tamoxifen. Subsequently, in 1992 the Early Breast Cancer Trialists’ Collaborative Group (EBCTCG) [2] published their meta-analysis of adjuvant studies which demonstrated a 39% reduction in contralateral breast cancers in women taking tamoxifen (SD 9, 1p < 0.0001). Two prevention trials, NSABP-P1 [3] and IBIS-1 [4] further confirmed tamoxifen’s inhibitory effect on ER positive tumours but no effect on ER negative disease. Prichard *et al.* [5] concluded that the women deriving the greatest benefit from tamoxifen are premenopausal with a five year GAIL risk factor of >1.66%, postmenopausal women with a GAIL risk factor of more than 3% and postmenopausal without a uterus. The MORE study [6] in postmenopausal women with osteoporosis, who took placebo or raloxifene, reported that there was a 76% decrease in all breast tumours, specifically of ER positive phenotype (95% confidence interval [CI], 0.13–0.44; P < 001). Bennett *et al.* [7] demonstrated elevated levels of prostaglandin in human breast cancer cells, making clear a potential role for NSAIDS in breast cancer chemoprevention. 

Epidemiological studies of association between non-steroidal anti-inflammatory drugs, such as ibuprofen and aspirin, and breast cancer risk have yielded inconsistent results. NSAIDs are non prescription medications used to manage the pain of many diseases including musculoskeletal disease and osteoarthritis in the elderly, and aspirin in the prevention of cardiovascular disease. Because of co-morbidity these indications for use are more common in the elderly population.

There is evidence, predominantly epidemiological but also experimental, that NSAIDS have a chemo preventative role in cardiovascular disease and in certain malignancies, with the most evidence in colorectal cancer.

## 2. Evidence of NSAIDS Role in Cancer Prevention

Evidence shows a chemo preventive effect for aspirin and other NSAIDS in colorectal cancer however the risk-benefit profiles for cancer prevention are insufficient and currently no definitive recommendations have been made. Aspirin is the most likely candidate for chemoprevention due to its known cardiovascular benefit and longer term safety and efficacy data. [8] The current literature does not agree on the dose and duration of aspirin, ibuprofen and selective COX-2 inhibitors, indicating the need for more research. 

The greatest evidence base for the role of these drugs in cancer chemoprevention is colorectal cancer. Sulindac and selective COX-2 inhibitors such as celecoxib have been given to patients at high risk of colorectal cancer, for example those with Familial Adenomatous Polyposis syndrome, this disease having provided an excellent study population. It is only aspirin that provides cardiovascular benefit. There have been numerous problems with cancer chemoprevention trials using COX-2 inhibitors. The APPROVE trial randomised patients with colonic polyps to rofecoxib or placebo, but in 2004 this trial was stopped due to significant cardiovascular side effects. The drug was then withdrawn from use worldwide. In the APC (Adenoma Prevention with Celecoxib) trial there was a 2.5 fold increase in the risk of fatal or non-fatal cardiovascular events and again after two years the trial was halted [9]. The preSAP trial [10] showed no difference in cardiovascular risk when patients were given placebo or celecoxib, although this was half the dose of celecoxib compared to that used in the APC trial.

Based upon meta-analysis of the published epidemiological evidence, use of either selective or non-selective COX -2 inhibiting agents significantly reduced the risk. Regular intake (of primarily 325 mg aspirin or 200 mg ibuprofen taken two or more times per week for at least 5 years) produced composite risk reductions of 43% for colorectal cancer, 24% for breast cancer, 28% for lung cancer and 27% for prostate cancer. A further recent series of case-control studies designed to compare selective and non-selective COX-2 inhibitors, daily intake of selective COX-2 inhibitors (20 mg celecoxib or 25 mg rofecoxib) produced a 70% composite risk reduction across all four major malignancies compared to 60% for ibuprofen (non-selective) and 54% for aspirin ( relatively selective COX-1 inhibition) [11]. 

## 3. Cyclo-Oxygenases, Cancer and Mechanism of NSAIDS

NSAIDS inhibit cyclooxygenase (COX) activity and subsequently reduce prostaglandin synthesis. Prostaglandins increase aromatase gene expression thereby stimulating estrogen biosynthesis in fat and muscle [12]. NSAIDS act by inhibiting of COX synthesis, which is the rate-limiting enzyme for prostaglandin biosynthesis. There are two genetic isoforms of COX. COX-1 is constitutively produced in most tissues and COX-2, the inducible form induced by pro-inflammatory stimuli [13]. Molecular studies have highlighted a role for COX-2 over expression in tumourogenesis, including breast cancer. COX-2 over-expression is a critical component in angiogenesis, mutagenesis, inhibition of apoptosis and aromatase-catalysed oestrogen biosynthesis together with immunosupression [14,15] thus stimulating tumour growth [16]. High levels of COX-2 expression are evident throughout tumourogenesis in every anatomic site that has been examined [17]. Studies have observed that COX-2 over-expression is sufficient to transform normal cells to malignant neoplasms in models of carcinogenesis [18]. COX-2 is not usually detectable in normal tissues, except the testes and brain.

This process of carcinogenesis involving highly selective cellular pathways and molecular changes in response to the over-expression of COX-2 and this prostaglandin cascade is often referred to as the “inflammogenesis of cancer”. COX-2 over-expression has been detected in approximately 40% of invasive breast cancer by immunohistochemistry and correlates with aggressive factors in breast cancer, including large tumour size, high histological grade, ER/PR negative and Herceptin (HER-2) over-expression [19,20]. COX-2 over-expression is associated with poor survival in ER/PR positive tumours but not ER/PR negative ones [21]. Use of NSAIDS has been shown to improve the survival of breast cancer patients [22]. NSAIDS, which include the non-selective aspirin and ibuprofen (COX-1 and COX-2 inhibitors) and COX-2 inhibitors (celecoxib) suppress the COX activity and reduce prostaglandin synthesis. Prostaglandins have organ-site-specific effect of increasing the levels of peripheral aromatase which stimulates oestrogen and progesterone biosynthesis [23], hence the theory behind their chemo preventative role in breast cancer. Thun *et al.* found evidence that NSAIDs may have the ability to restore apoptosis and inhibit angiogenesis, which would have an obvious therapeutic value.

Two studies in rats identified the chemoprevention of breast cancer by the selective COX-2 inhibitors, rofecoxib and celecoxib [24,25]. There is considerable evidence to suggest that COX inhibitors alone or in combination with other agents are useful in the chemoprevention of breast cancer [26]. The COX-2 inhibitor celecoxib has been shown to inhibit *in vitro* growth of MDA-MB-231 breast cancer cell and this cell line is estrogen receptor negative suggesting that celecoxib maybe a potential agent for the prevention of ER negative tumours. Celecoxib is a potential agent for breast cancer chemoprevention, alone or in combination with an aromatase inhibitor [27].

## 4. Relationship between COX-2 and Aromatase.

This relationship has a potential role in preventing breast cancer in older women maybe taking NSAIDS for musculoskeletal disease such as osteoarthritis and/or aspirin for cardiovascular disease prevention, that are ER positive, through inhibiting oestrogen via NSAIDS.

Experimental and epidemiological evidence suggests a significant role for the COX enzymes, particularly COX-2 in the pathogenesis of breast cancer [28]. It is known that prostaglandin E2 increases aromatase gene expression and that the synthesis of prostaglandins is catalysed by COX enzymes. Animal studies have demonstrated that aromatase activity is suppressed in COX-2 knockout mice and aromatase activity is increased by transgenic COX-2 over-expression [20,29]. COX inhibitors decrease aromatase activity in breast cancer cells and this effect starts at the transcriptional level. Real time PCR data shows that in this mechanism, promoters 1.4 and II are involved in breast cancer development. Thus prostaglandin E2 produced via COX may act locally in paracrine and autocrine fashion to increase the biosynthesis of estrogen by aromatase in hormone dependent breast cancer development [30]. There is therefore a theoretical possibility that NSAIDS will impair this sequence of COX induced PG expression and thus reduce aromatase gene expression, reducing the level of estrogen and therefore breast cancer risk. A selective COX-2 inhibitor having greater efficacy.

## 5. Ongoing Clinical Trials Involving Celecoxib

NCIC Clinical Trials Group phase III trial of examestane with or without celecoxib *versus* placebo in high risk postmenopausal women, are awaited with interest. REACT (Randomised European Celecoxib Trial) is a multicentre, placebo controlled, randomized phase III trial in patients with primary breast cancer. Patients, in addition to their standard adjuvant therapy, are randomized between two years celecoxib (a COX-2 inhibitor) and placebo in a 2:1 ratio in favour of celecoxib. This trial is still recruiting patients.

## 6. Evidence of Breast Cancer Prevention by NSAIDS

In 2009 a meta-analysis and systematic review was executed by Zhao *et al.* [31] to explore the pooled relative risk and 95% confidence intervals of the epidemiological studies on breast cancer risk and NSAIDS. They included 26 studies with 528,705 participants. The Relative Risk (RR) of NSAID use and incidence of breast cancer was 0.94 (95% CI: 0.88–1.00) with random effects model. A slight reduction of breast cancer by taking aspirin and ibuprofen was both observed with pooled RR of 0.91 (95% CI: 0.83–0.98) and 0.81 (95% CI; 0.67–0.97) respectively. These indicate that non-selective NSAID use is associated with a slight decrease for the development of breast cancer with a marginally statistically significant difference. 

Another meta-analysis published in 2001 by Khuder *et al.* [32] and another in 2008 by Mangiapane *et al.* [33] demonstrated pooled RR of 0.82 (95% CI: 0.75–0.89) and 0.75 (95% CI: 0.64–0.88) respectively. There was significant heterogenecity in these studies and no clear comparison on dose and frequency of the NSAIDs. One Danish cohort study involving 28,695 women actually showed an increase risk of breast cancer compared with no use of the drugs, RR 1.27 (95% CI: 1.10-1.45) [34]. In Zhao et al’s meta-analysis from 2009 [31], sub group analysis showed the breast cancer risk reduction was higher in the case control studies (12%) than the cohort studies (4%) but was not statistically significant. This overview also highlighted three papers investigating the long-term use (>10 years) of NSAIDS none of which showed a decrease in breast cancer development. Daily and no less than four times per week of NSAID intake were associated with risk reduction in breast cancer.

Experimental studies have also analysed the association of breast cancer risk, chemoprevention and NSAID usage. Another mechanism is the nitric oxide pathway which has anti-cancer properties by way of its anti-oxidant properties.

A study by Khalkhali-Ellis *et al.* [35] demonstrated that NO exerts its anticancer properties *in vitro* and increases production of maspin, which is inhibitory in malignant breast cancer cells. Girish *et al.* [36] treated 15 breast cancer patients and 15 age-matched controls with 150 mg daily of aspirin and reported there was both an increase in serum nitric oxide and maspin levels in the breast cancer patients. This however was a very small study population making extrapolation of results very difficult. 

Nitric oxide donating aspirin (NO-ASA) consisting aspirin and an -ONO2 moiety linked to it via a molecular spacer is a new drug mechanism for cancer prevention. NO-ASA inhibits a number of cancers including breast cancer and is up to 6,000x more potent than traditional aspirin, suggesting a promising agent for the future prevention and/or treatment of cancer [37]. A recent study of DNA-adducts in urine demonstrates that women at high risk of, or with breast cancer, have high levels, indicating a critical role for adduct formation in breast cancer initiation. This knowledge of the first step in cancer initiation suggests it would be possible to use specific anti-oxidants that can block formation of the adducts by chemical and biochemical mechanisms. Two anti-oxidants, *N*-acetylcysteine and revesterol are prime candidates to prevent breast cancer because they reduce the formation of oestrogen-DNA adducts in various *in vitro* and *in vivo* experiments [38].

## 7. NSAIDS and *In-Situ* Disease

COX-2 is commonly found in pre-malignant lesions, carcinoma *in situ*, invasive cancer and metastatic disease. High levels of expression of COX-2 are observed in premalignant lesions such as atypia of the mammary gland [39] COX-2 over-expression accounts for 63–80% of cases in DCIS [40] The North Carolina Breast Cancer Study reported a reduced incidence of *in-situ* disease in women taking NSAIDs compared with controls (OR 0.7 95% CI, 0.4–1.1) This association was weaker than invasive disease and not statistically significant.

## 8. Stage of Breast Cancer and Hormone Receptor Status

In 2004, a case control study with 1,442 cases and 1,420 controls reported a reduction in risk of breast cancer with aspirin use among those women with hormone receptor positive tumours (OR = 0.74, 95% CI = 0.60) but not for women with hormone receptor negative tumours (OR = 0.97, 95% CI = 0.67–1.40) [12].

The California Teachers study [16] study concludes daily use of NSAIDS was not associated with breast cancer risk overall. Ibuprofen use was associated with an increased risk of breast cancer (RR = 1.51, 95% CI = 1.17–1.95), and long term daily aspirin use was associated with increased risk of ER/PR negative breast cancer (RR = 1.81, 95% CI = 1.12–2.92) but it is not clear if this is causal. Among postmenopausal women, an inverse association with aspirin was seen among hormone rector positive tumours and not with hormone receptor negative tumours. 

Among premenopausal women there was no statistically significant association in both hormone receptor positive and negative tumours. Other studies have highlighted that the association of NSAIDS and reduced breast cancer risk was not significantly different according to hormone receptor status [41,42]. A study by McCarthy in 2006 [43] found a significantly higher expression of COX-2 mRNA in ER and PR negative cancers, suggesting a role for COX-2 inhibitors in ER negative breast cancer patients.

A nested case control study by Sharpe *et al.* [44] was interpreted as showing that NSAIDs are unlikely to prevent breast cancer from developing *de novo* but might slow the growth of tumour, reducing the probability of detection clinically and radiologically. They highlighted protective effects of NSAIDS on incidence, tumour size and distant metastases and all associated with exposure 2–5 years before diagnosis. There was no effect on incidence of regional lymph node metastases. 

## 9. COX-2 Inhibitors in Combination with Aromatase Inhibitors in Metastatic Breast Cancer

Aromatase inhibitors (AI) and COX-2 (celecoxib) inhibitors have shown promising efficacy and safety in the treatment for patients with metastatic breast cancer. Combination therapy had better or comparable efficacy compared with AI monotherapy using the end-points of progression free survival, overall response rate and, clinical benefit rate, time to progression and duration of clinical benefit, with therapies being well tolerated. There also appeared to be beneficial impact on serum lipid levels for patients receiving combination therapy in a neo adjuvant trail despite the known cardiovascular risk associated with COX-2 inhibitors. This highlights a therapeutic role of COX-2 inhibitors in breast cancer [45].

## 10. Age and Breast Cancer in Older Women

As the population as a whole now has a longer lifespan, the incidence of certain age-related cancers, such as breast cancer, will also increase. Some studies by Diadone, Gennari and another by Fischer in 1997 [46,47,48] have shown that older patients had tumours with more favourable biological features such as a lower proliferation rate, normal p53 and higher expression of oestrogen and progesterone receptor status and the proportion of ER positive tumours in older women being more than 80%.

Age related incidence of breast cancer in general is due to breast tissue aging rather than patient chronological age [49]. Also age itself is associated with failure of DNA repair mechanisms and is thus a major risk factor for all solid tumours. The problem however, with long term NSAID use in the elderly is the risk of NSAID related complications. These include gastrointestinal abnormalities such as peptic ulcer disease leading to GI bleeding and perforation, this can be counteracted with the concomitant use of a proton pump inhibitor. 

Johnson *et al.* [50] performed a six year study of aspirin and other NSAID use in a cohort of 27,616 post-menopausal women with a reported risk reduction of 20% in the overall group but the benefit appearing to be with aspirin alone [(RR) 0.80, 95% confidence interval (CI) 0.67–0.95].

A study by Sauter *et al.* [51] collected nipple aspirate fluid (NAF) from a small number of women (26), at increased risk of breast cancer. Both NAF and plasma samples were taken before starting celecoxib, two weeks later after 14 days of celecoxib 200 mg twice daily and then two weeks after stopping treatment. Prostaglandin E2 levels in the NAF and serum were unchanged among pre-menopausal women. In contrast among the postmenopausal women at high risk there was significant reduction in PGE2 after celecoxib.

## 11. Conclusions

As has been shown, there are many potential benefits for the use of selective and non-selective NSAIDS in all aspects of breast cancer chemoprevention, along with treatment benefits in metastatic disease in combination with aromatase inhibitors. There remains a great deal of work to be done to lose the heterogenecity of the current data, especially in relation to the dose and frequency of aspirin and ibuprofen. NSAIDs have a potential significant chemoprevention role against the development of breast cancer but more clinical research is required, especially with side effect profiles of these drugs. The mortality risk and morbidity associated with these drugs would need to be carefully assessed prior to regular use in chemoprevention of breast cancer.

Epidemiological evidence suggests that non-steroidal anti-inflammatory drugs (NSAIDs) which act as COX-2 inhibitors may reduce breast cancer incidence by 20%. Once again there is no consistent evidence on dose and/or frequency and duration of such drugs. NSAIDs are also used for the treatment of osteoporosis which it the most common form of arthritis occurring in 40% of women aged between 70 and 74 years. The incidence of breast cancer increases with age thus providing a potential opportunistic method of breast cancer chemoprevention with the use of NSAIDS [52]. The future still needs to be defined. 

NSAIDs reduce serum estradiol by 17% in post-menopausal women. Most post-menopausal women who develop breast cancer have estrogen receptor positive tumours, suggesting a preventative role for NSAIDs. Careful use of these agents could provide a strategy for both relief of symptoms of osteoarthritis and also breast cancer prevention. Instead of conducting a randomised trial, proof of efficacy could be from an adequately powered cohort study within the breast screening programme.
